# Subtenon Vs Intravitreal Triamcinolone injection in Diabetic Macular Edema, A prospective study in Chinese population

**DOI:** 10.12669/pjms.304.4810

**Published:** 2014

**Authors:** Dawei Luo, Bijun Zhu, Zhi Zheng, Haidong Zhou, Xiaodong Sun, Xun Xu

**Affiliations:** 1 Dawei Luo, Nanjing Medical University, Nanjing, Jiangsu Province, 210029, China.; 2 Bijun Zhu, Department of Ophthalmology, Shanghai First People’s Hospital, Shanghai, 200080, China.; 3 ZhiZheng, Department of Ophthalmology, Shanghai First People’s Hospital, Shanghai, 200080, China.; 4 Haidong Zhou, Department of Ophthalmology, Shanghai First People’s Hospital, Shanghai, 200080, China.; 5 Xiaodong Sun, Department of Ophthalmology, Shanghai First People’s Hospital, Shanghai, 200080, China.; 6 Xun Xu, Nanjing Medical University, Nanjing, Jiangsu Province, 210029, China.

**Keywords:** Diabetic Retinopathy, Intravitreal, Macular Edema, Subtenon, Triamcinolone

## Abstract

***Objective:*** Purpose of this study was to validate that Subtenon (SB) Triamcinolone (TA) injection is an alternative to Intravitreal (IV) Triamcinolone (TA) injection for the treatment of diabetic macular edema (DME).

***Methods:*** Forty eyes were selected having DME due to type 1 or type 2 diabetes. All the patients were treated with photocoagulation. IVTA was administered in one eye and SBTA in following eye of same patient. Improvement in visual acuity, macular edema and intraocular pressure was assessed before treatment and on 2^nd^, 4^th^, 8^th^ and 12^th^ week after treatment.

***Results:*** After administration of IVTA, MVA was reduced from baseline value (0.805 ± 0.069Log/MAR) to (0.577 ± 0.091 Log/MAR, p<.001) at the end of treatment. Similar results were observed after SBTA administration. MVA was reduced from (0.814 ± 0.082Log/MAR) to (0.49 ± 0.080 Log/MAR, p<.001) at 12^th^ week. After IVTA injection Central macular thickness was significantly reduced to (246.8 ± 25 µm, p<0.001) from (390.5 ± 17 µm). There were no significant (p=0.51) difference in both eyes receiving different routes of same treatment. After SBTA injection CMT was significantly reduced to lower values (241.5 ± 27 µm, p<0.001) from (394.4 ± 21 µm). Intraocular pressure after IVTA administration was high (2.32 ± 0.72 mm/Hg, p=0.04) as compared to baseline (1.82 ± 0.94 mm/Hg). Similar pattern was also seen after SBTA administration but to significant extent. Elevation of IoP was observed in both eyes.

***Conclusion:*** Subtenon Triamcinolone injection is an alternative to Intravitreal Triamcinolone Injection for Diabetic Macular Edema.

## INTRODUCTION

According to a survey 3.2% of Chinese population is suffering from Diabetes.^1^ Diabetic retinopathy (DR) is sequelae of diabetes that can eventually lead to blindness.^1^ Its incidence is about 80% in patients who have had diabetes for 10 years or more but its progression and incidence can be reduced with vigilant treatment and monitoring of eyes.^2^ Diabetic patients have 29 fold risk of developing blindness as compared to non-diabetic patients.^3^ DR ranges from non-proliferative (NPDR) to progressive proliferative DR (PDR) and blindness is caused by macular edema, retinal ischemia, retinal fibrosis, vitreous hemorrhage and retinal detachment. Diabetic Macular Edema (DME) is characterized by microanuerysms and hard exudates that can be detected by stereoscopic examination techniques (SET). DME is a major cause of vision loss in patients and it can occur at any stage of disease. Patients having duration of T1DM 5 years and 20 years have incidence 0% to 29% and in case of T2DM ranges from 3% to 28% in same duration respectively.^6^ Retinal thickness at or within 500 um is termed as clinically significant macular edema.^4^ Intensive diabetic therapy can reduce incidence of NPDR and PDR by 47%.^5^

Laser photocoagulation^7^, virectomy, intravitrials injections of anti-vascular endothelial growth factors (VEGF) and triamcinolone acetonide are different available options for DME.^8^^-^^10^ Intravitrial Triamcinolonacetonide (IVTA) 4mg is considered primary or adjuvant therapy but dose may ranges from 4-25 mg.^11^ IVTA is a promising treatment for DME^12^ but inexicably linked to elevation of intraocular pressure^13^, retinal detachment, glaucoma, ocular hypertone, intraocular hemorrhage endophthalmitis^14^ and Subtenon triamcinolone.^15^^-^^17^

Considerable efforts have been made to compare these both routes in DME but is very limited data available on Chinese Population.^18^ So we conducted a prospective comparative study to validate the statement the IVTA and SBTA are equally effective and are alternatives in Chinese patients suffering from DME.

## METHODS

Total 20 patients (40 Eyes) were selected for this prospective study. Male to female ratio was 3:1. Mean age of the patients were 64.7 years suffering from type 2 or type 1 diabetes mellitus.

Phackic eyes, without retinal vitreous traction, age > 18 years, previously treated with laser photocoagulation and macular thickness > 250 um on optical coherence tomography (OCT) were inclusion criteria of this study. Patients having ocular trauma or surgery, laser or cataract surgery within 3 or 6 months of recruitment respectively, PDN, glucouma, ocular hypertension, history of uveitis, endophalmitis and extensive foveal ischemia were excluded from this three months months observational study and patients were recruited from Department of Ophthalmology, Shanghai First People’s Hospital, Shanghai, 200080, China. Patient’s recruitment and treatment duration was March 2011 to May 2013. All the patients were informed about the purpose and protocol of the study and written consents were taken from them on approved Performa. Protocol approval was taken from Ethical Review Board of Shanghai First People’s Hospital, Shanghai, 200080, China, by providing documents having protocol of study and informed patient’s consents Forms. 

Macular thickness, intro-ocular pressure (IOP) and minimum angle of resolution visual acuity (LogMAR) were observational parameters that were determined by optical coherence tomography (OCT), tonometry and early treatment Diabetic Retinopathy Studies (ETDR) charts respectively.

**Fig.1:**
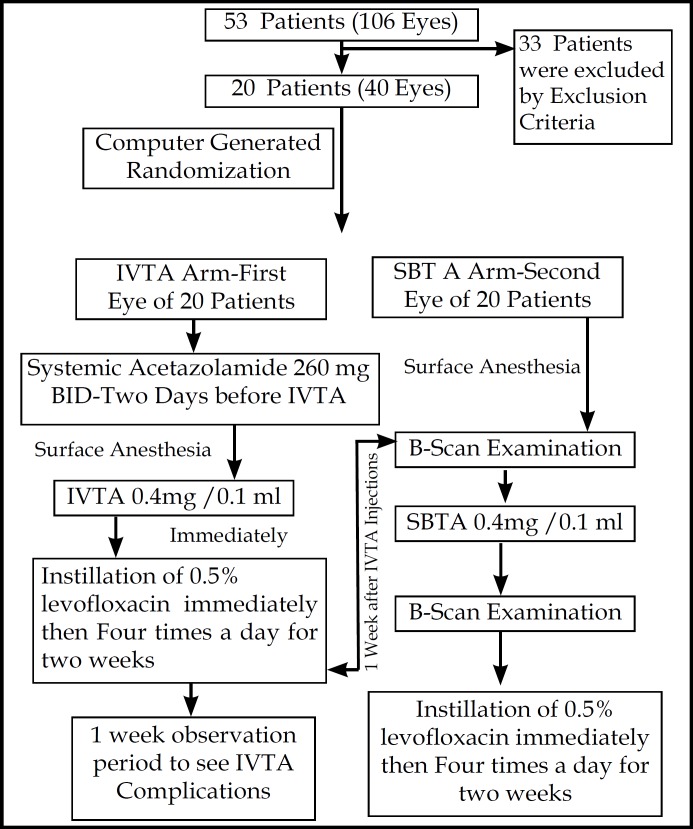
Study methodology

IVTA and SBTA injections were administered in supine position of patients by using 30 gauge and 27 gauge (2.5 ml syringe) needles respectively. Firstly IVTA was administered in one eye and waited to observe any complication for one week, then SBTA was administered in fellow eye and B-Scan was performed before and after administration of SBTA to ensure deposition of Injection contents in macular region. Before injections 4% carbocaine followed by 5% povidine iodide and 0.4% oxybuprocaine were applied as surface anesthetics for IVTA and SBTA respectively. Ophthalmic antibiotics were prescribed after each injection. All the injections were given in strict aseptic conditions. IVTA injection was performed by one author and SBTA was by other. The physicians who observed IOP, CMT and visual acuity were not informed about the study and drugs given to the patients. Study methodology is shown in Fig.1. 

All the patients were evaluated for observational end points before injection and on 2nd week, 4^th^ week, 8^th^ week and 12^th^ week (3 months observation) of post injection. CMT was determined by OCT and six radial scans were taken for each eye. Visual acuity was determined by recommended charts and converted to logMAR by using statistics. IOP was determined by Golmannapplanation tonometry. All the medications used during study period were recorded e.g. for Glaucoma. (Fig.1)

All the values are represented in means ± SD (standard deviation) and were subjected to statistical analysis by using One Way ANOVA (Minitab version 16.1.0.0 Statistical Software) and probability values p<0.05 were considered as statistical significant.

## RESULTS

**Fig.2:**
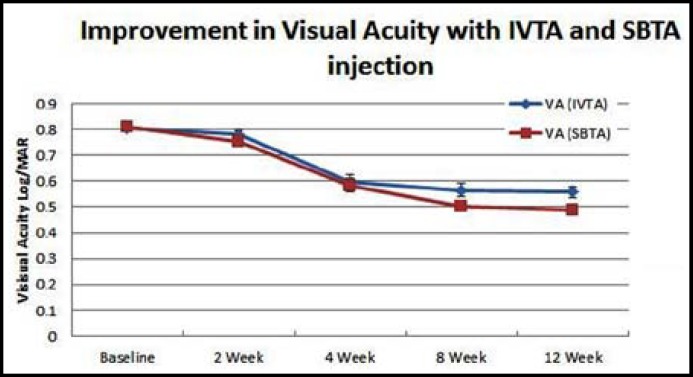
Improvement in visual acuity with IVTA and SBTA injection

The Mean visual acuity (MVA) was evaluated before treatment (baseline) and then after 2, 4, 8 and 12 week. Both routes provide significant reduction (p < 0.001). After administration of IVTA, MVA was reduced from baseline (0.805 ± 0.069 Log/MAR) to (0.577 ± 0.091 Log/MAR, p<.001). Vision improvement was observed at the end of observation period and was confirmed by asking visual satisfaction from patients. By the same way similar results were observed after SBTA administration. MVA was reduced from baseline (0.814 ± 0.082 Log/MAR) to (0.49 ± 0.080 Log/MAR, p<.001). There were no significant difference (p > 0.05) between two routes till 8^th ^week of treatment but MVA was increased in IVTA arm while this reduction of MVA was maintained by SBTA injected eye. MVA before and after 2^nd^, 4^th^, 8^th^ and 12^th^ week of IVTA and SBTA are given in Table-I and graphically represented by Fig.2.

**Fig.3:**
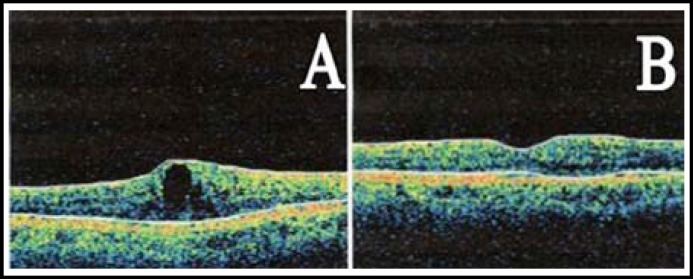
Optical coherence tomography (OCT) Images of eye treated with SBTA injection showing macular thickness before and after treatment

After IVTA injection CMT was significantly reduced (246.8 ± 25 µm, p<0.001) as compared to baseline (390.5 ± 17 µm). There were no significant (p=0.51) difference of MT in both eyes receiving different routes of same treatment. After SBTA injection CMT was also significantly reduced to (241.5 ± 27 µm, p<0.001) from (394.4 ± 21 µm). There were no significant difference (p ≥ 0.5) between two treatment eyes but individual observations showed macular thickness improvement was becoming reverse but not by significant extent while SBTA eyes lack this reversal of effect. Macular thickness before and after IVTA and SBTA injections are given in Table-II. Fig.3 represents OCT Images of eye treated with SBTA injection showing macular thickness before and after treatment.

The mean intraocular pressure (IoP) before triamcinolone acetonide injection and after 2^nd^, 4^th^, 8^th^ and 12^th^ week are showed in Table-III. Mean IoP was equivalent in both eyes (p=0.94) receiving SBTA and IVTA injections in same conditions and methods. Iop after IVTA administration was high (2.32 ± 0.72 mm/Hg, p=0.04) as compared to baseline (1.82 ± 0.94 mm/Hg) showing that IVTA injections caused increase in intraocular pressure. Iop after SBTA administration was high (1.90 ± 0.80mm/Hg, p=0.83) as compared to baseline (1.84 ± 0.88mm/Hg) showing that SBTA injection also caused increase in IoP but not to significant level as shown by IVTA injection. Elevation of IoP was observed in both eyes but more in eyes receiving IVTA injection. Graphical representation of intraocular pressure elevation is shown by Fig.5.

## DISCUSSION

This study was conducted to evaluate and validate that SBTA injection is an alternative or may be better than IVTA injection for the treatment of Macular edema which is among major causes of blindness. Forty eyes of 20 patients were examined for significant improvement of visual acuity and reduction of retinal thickness. Both eyes, one received IVTA and other SBTA injection, showed significant improvement in visual acuity score and central macular thickness. These results were significant until 3 months of treatment but on 12^th^ week reversal of visual acuity score and macular thickness were observed in eyes receiving IVTA injection. Eyes receiving SBTA the visual acuity and macular thickness were stable. Similar results have also been seen in other studies conducted, where on 6^th^ month of observation patients receiving IVTA showed significant reduction in visual acuity score and increase in macular thickness.

**Table-I:** Visual acuity before and after IVTA and SBTA triamcinolone injection

***Mean Visual Acuity (MVA) log/MAR***	***IVTA (Mean ± SD)***	***SBTA (Mean ± SD)***	***p value (b/w IVTA and SBTA)***	***p value (IVTA and Baseline)***	***p value (SBTA and Baseline)***
Baseline	0.805 ± 0.069	0.814 ± 0.082	p = 0.72		
2 Weeks	0.781 ± 0.019	0.75 ± 0.068	p = 0.20	p < 0.27	p < 0.01
4 Weeks	0.598 ± 0.128	0.58 ± 0.115	p = 0.71	p < 0.001	p < 0.001
8 Weeks	0.564 ± 0.117	0.50 ± 0.090	p =0.07	p < 0.001	p < 0.001
12 Weeks	0.577 ± 0.091	0.49 ± 0.080	p <0.01	p < 0.001	p < 0.001

Results are presented as Mean ± SD. The probability values p<0.05, p<0.01 and p<0.001 are considered as significant.

**Table-II:** Macular thickness before and after IVTA and SBTA triamcinolone injection

***Central Macular Thickness (CMT)***	***IVTA (Mean ± SD) µm***	***SBTA (Mean ± SD) µm***	***p value (b/w IVTA and SBTA)***	***p value (IVTA and Baseline)***	***p value (SBTA and Baseline)***
Baseline	390.5 ± 17	394.4 ± 21	p = 0.51		
2 Weeks	302.8 ± 27	299.1 ± 23	p = 0.30	p < 0.001	p < 0.001
4 Weeks	262.9 ± 26	254.8 ± 24	p = 0.31	p < 0.001	p < 0.001
8 Weeks	242.1 ± 22	240.5 ± 23	p = 0.83	p < 0.001	p < 0.001
12 Weeks	246.8 ± 25	241.5 ± 27	p = 0.50	p < 0.001	p < 0.001

Results are presented as Mean ± SD. The probability values p<0.05, p<0.01 and p<0.001 are considered as significant

**Table-III:** Intraocular Pressure (IoP) before and after IVTA and SBTA triamcinolone injection

***Intraocular Pressure (IOP)***	***IVTA (Mean ± SD) mmHg***	***SBTA (Mean ± SD) mmHg***	***p value (b/w IVTA and SBTA)***	***p value (IVTA and Baseline)***	***p value (SBTA and Baseline)***
Baseline	18.2 ± 1.94	18.4 ± 1.88	p = 0.94		
2 Weeks	19.1 ± 2.81	18.7 ± 2.76	p = 0.88	p = 0.76	p = 0.91
4 Weeks	20.3 ± 3.69	18.8 ± 2.77	p = 0.52	p = 0.42	p = 0.87
8 Weeks	22.2 ± 2.73	18.9 ± 3.79	p = 0.17	p = 0.14	p = 0.85
12 Weeks	22.6 ± 2.72	19.0 ± 3.80	p = 0.08	p < 0.04	p = 0.83
Results are presented as Mean ± SD. The probability values p<0.05, p<0.01 and p<0.001 are considered as significant. (Baseline: 0 Week, before treatment)					

**Fig.4:**
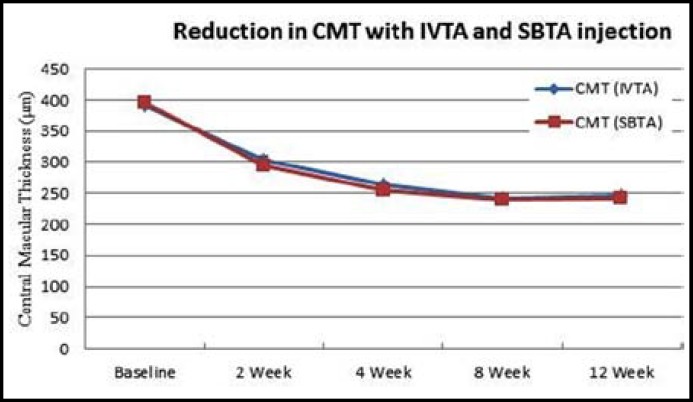
Reduction in CMT with IVTA and SBTA injection

**Fig.5:**
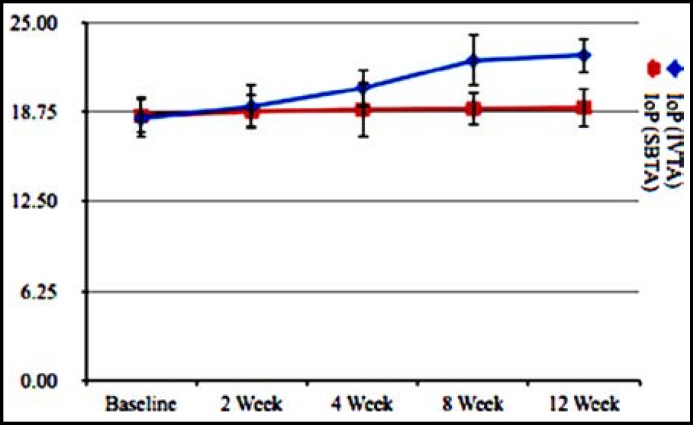
Elevation in intraocular pressure with IVTA and SBTA injection

Rise in intraocular pressure (IoP) is a disadvantage of IVTA injection requiring anti-Glaucoma treatments that was observed at 2, 4, 8 and 12 weeks of treatment. Final observation of patients having IVTA showed marked elevation in intraocular pressure as compared to baseline while on other in SBTA injecting eyes rise in IoP was also observed but not in significant pattern. Insignificant elevation of IoP in SBTA patients required no treatments for Glaucoma as compared to IVTA patients among which some were taking medications for reduction of IoP.

In this study improvement in Visual Acuity after 2, 4, 8 and 12 weeks were 3.1%, 25.8%, 30% and 29.9% respectively after IVTA injection and 7.1%, 28%, 37.8% and 39.6% after SBTA injection. Visual acuity improvement is better in eyes receiving SBTA as compared to IVTA eyes. SBTA route superiority can`t be claimed on this basis as this route also required B-Scan to evaluate proper placement of drug in Macular bed. This superiority can be claimed after large randomized trial having same objective. Effectiveness of SBTA injection has been established for Cystoid Macular Edema, Uveitis and Diffuse Macular Edema refractory of Virectomy. In contrast it has also been observed that SBTA administration provides sustained and stable results of visual acuity and vision improvement up to 12 months.^19^^-^^21^ In another comparative study it was concluded that IVTA is better than SBTA for anatomical improvement of retina.^22^ Some studies also claimed that IVTA injections are better for treatment of refractory diffuse macular edema as compared to SBTA. These two studies have results that are opposite to our findings that both SBTA and IVTA are equally effective for short term treatment. 

Steroid use in edema is important because of its inhibition of Arachidonic acid cascade pathway, down-regulation of cytokines and avoiding tearing of hemato-reinal barrier. All predisposing factors that cause edema in diabetic patients such as increase production of prostacyclins, vascular endothelial growth factor, and macrophages cellular component, Cyclooxygenase 2 and prostacyclin synthase are reduced with the administration of Intravitreal or Subtenon Steroids.

Macular thickness after 2, 4, 8 and 12 weeks were reduced by 22.5%, 32.7%, 38.1% and 36.8% respectively after IVTA injection and 26.5%, 35.4%, 39.1% and 38.9% after SBTA injection. These results also related to previous findings of equally effectiveness of Intravitreal and SubTenon routes for Macular edema treatment. Our findings also related these results with previous studies on different population. For Macular thickness reduction SBTA provides greater reduction in thickness but not in significant manner as compared to IVTA. Photocoagulation is a gold standard for the treatment of Macular edema but it can`t restore visual loss rather than preventing further visual loss up to 50%. Photocoagulation has also been reported to cause edema during treatment and lastly its compromised efficacy for diffuse macular edema opened new options for the treatment of Macular Edema. Triamcinolone acetonide can be a good option for such patients (Early Treatment Diabetic Retinopathy Research Group).

Visual acuity and central macular thickness improvement sustain for only three months after which visual acuity score increased towards blindness and macular thickness also increases to higher values. It may be due to different complications of Intravitreal route e.g. Rise in Intraocular pressure, endophthalimitis, retinal detachment and intraocular hemorrhages. Intravitreal TA is only effective for short term treatment that’s why IVTA injection is repeated at three months interval to maintain macular anatomy and retinal physiology.^23^

Elevation of Intraocular pressure in a complication during IVTA therapy that sometimes requires medications for cataract. That’s why most patients suffering from macular edema due to diabetic retinopathy take anti-glaucoma drugs for its management. Increase in IoP was 21.5% after IVTA injection and 3.0% after SBTA injection. 20-80% increase in intraocular pressure has also been observed in previous studies. These results suggest the lower incidence of Glaucoma hence medication use in patients receiving SBTA injection. Same findings were also observed in previous studies but our study provides good results as compared to previous one. It may be due to Genetic variation of Chinese Population but genetics are beyond scope of our study so we simply conclude that SBTA injections are better than IVTA injections as regards increase in Intraocular Pressure. Endophthalmitis and retinal detachment are also among complication of Intravitreal route that were observed in some patients during our study either.

As both routes have complications “less or more” with advantage of less increment in intraocular pressure with Subtenon route so we are not in a position to declare Subtenon route as a safe and better option than Intravitreal route and that was not purpose of our study.

## CONCLUSION

In Conclusion, the short term effectiveness of IVTA and SBTA injections for improvement in Visual Acuity and Macular edema is equal. Besides these Subtenon route has advantage over Intravitreal route that it doesn’t significantly increase intraocular pressure to such extent that requires medication. Less invasiveness and safety are other added on benefits of Subtenon injection over Intravitreal route. Subtenon route in common for anesthetic injection for cataract surgery. By this route conjunctiva opening can be avoided thus it improves patient compliance. Echographic examination is required in this route to ensure proper placement of steroid in Subtenon region. Hence optimization of good results after Subtenon injections depends upon proper placement of drug in macular bed. Subtenon route adds some technicality with apparatus use for scanning purpose. But this simple looking method also has different complications such as accidental injection into choroidal and retinal circulation, Ocular Bulb perforation, cataract and conjunctiva necrosis.^24^ Our results suggest Subtenon route is an alternative to that of Intravitreal route for administration of steroid in patients suffering from cystoid or diffuse macular edema with glaucoma or with other complications that avoid Intravitreal route. Proper placing of steroid in macular bed is necessary to obtain good results otherwise injection can cause more complications. This limited Chinese patients study suggests Subtenon injection is a safe, effective and easy alternative to that of Intravitreal route.
